# Efficacy analysis of one-stage posterior-only surgical treatment for thoracic spinal tuberculosis in the T4–6 segments with minimum 5-year follow-up

**DOI:** 10.1038/s41598-021-04138-2

**Published:** 2022-01-07

**Authors:** Yi Zhan, Xin Kang, Wenjie Gao, Xinliang Zhang, Lingbo Kong, Dingjun Hao, Biao Wang

**Affiliations:** 1 Department of Spine Surgery, Xi’an Jiaotong University College of Medicine, Honghui Hospital, Xi’an, 710054 Shaanxi China; 2 Shaanxi University of Chinese Medicine, Xi’an, 712046 China; 3 Department of Spine Surgery, Sun Yat-Sen Memorial Hospital of Sun Yat-Sen University, Guangzhou, 510120 China

**Keywords:** Tuberculosis, Orthopaedics

## Abstract

In recent years, with the in-depth research on spinal tuberculosis, posterior surgery alone has been praised highly by more and more surgeons due to the better correction of kyphosis, better maintenance of spinal physiological curvature, smaller surgical trauma and fewer surgical complications. However, there is currently lack of relevant reports about the efficacy of posterior surgery alone in the treatment of tuberculosis in the T4–6 segments. This study aimed to evaluate the clinical study efficacy and feasibility of one-stage posterior-only surgical treatment for thoracic spinal tuberculosis in the T4–6 segments. 67 patients with tuberculosis in T4–6 segments who underwent one-stage posterior-only surgery were included in this study. The clinical efficacy was evaluated using statistical analysis based on the data about erythrocyte sedimentation rate (ESR), C-reactive protein (CRP), Oswestry Dability Index (ODI) score, Visual Analogue Scale (VAS) score and Cobb angle before surgery, after surgery and at the last follow-up. All patients completed fusion during the follow-up period of 6–9 months. ESR and CRP were returned to normal for all patients at 6 months follow-up. In the meanwhile, among the 27 patients combined with neurological impairment, neurological functions of 22 cases (81.48%) recovered completely at the last follow-up (P < 0.05). Cobb angle of the kyphosis was improved from preoperative 34.8 ± 10.9° to postoperative 9.6 ± 2.8°, maintaining at 11.3 ± 3.2° at the last follow-up, The ODI and VAS scores were improved by 77.10% and 81.70%, respectively. This 5-year follow-up study shows that better clinical efficacy can be achieved for tuberculosis in T4–6 segments using one-stage posterior-only approach by costotransverse debridement in combination with bone graft and internal fixation. The posterior surgical method cannot only effectively accomplish debridement, obtain satisfactory clinical results, but also well correct kyphotic deformity and maintain it.

## Introduction

It has been suggested by the WHO (World Health Organization) 2019 global tuberculosis report that the burden of tuberculosis is still heavy on people all over the world. China, as the largest developing country in the world, ranks the second (9%) in the number of people with tuberculosis, which is also a great burden for China^1^. Spinal tuberculosis is the most common extrapulmonary tuberculosis, which is also the most common tuberculosis of bone joints, accounting for about 50%^2–4^. Although thoracic spinal tuberculosis is the most common spinal tuberculosis, lesions in the T4–6 segments are rare in the entire spinal tuberculosis. However, physicians should pay additional attention to this disease due to the clinical specificity of tuberculosis in these parts^5,6^.

T4–6 segments locate in the initial part of thoracic kyphosis, and the stress is concentrated, thus it is easy to cause the loss of spinal stability and kyphosis deformity after destroyed by lesions. Meanwhile, the spinal cord is susceptible to neurological damage due to compression of tuberculosis focus, which is harmful to the patients, seriously affecting the work and life of patients^7^. Additionally, the anatomical structures surrounding the T4–6 segments are complex, and location of the vertebral body is deep, making the lesions difficult to be exposed, and increasing surgical difficulty, which is a great challenge for the surgeons to design surgical strategy^8,9^. In order to solve these challenges, there are multiple surgical approaches in clinical practice such as anterior, posterior, or combined approaches. However, it is currently unclear which surgical approach is a better choice^10–12^. Anterior surgery can achieve direct debridement, decompression, bone graft and fusion, as well as reconstruction of spinal stability, which has significant advantages^10,13,14^. However, it has been reported that fixation via anterior approach alone may lack the ability of correcting kyphosis deformity and maintaining spinal curvature to avoid the progressive kyphosis after surgery^15,16^, thus the combination of anterior and posterior surgeries has been recommended by most scholars. However, it has the disadvantages of large surgical trauma, various complications and high cost^11,12,15^. In recent years, with the in-depth research on spinal tuberculosis, posterior surgery can obtain similar effects as the anterior surgery in debridement^2,17–19^. At the same time, it has been praised highly by more and more surgeons due to the better correction of kyphosis, better maintenance of spinal physiological curvature, smaller surgical trauma and fewer surgical complications^4,20^.

As far as we know, there is currently lack of relevant reports about the efficacy of posterior surgery alone in the treatment of tuberculosis in the T4–6 segments. This study was the first report with large sample size that used one-stage posterior-only surgery to treat tuberculosis in T4–6 segments. Purposes of this study were: (1). To retrospectively analyze the 67 patients with spinal tuberculosis in T4–6 segments who underwent one-stage posterior surgery by costotransverse debridement in combination with bone graft and internal fixation during the past 5 years follow-up in our center; (2). To investigate the clinical prognosis, safety and efficacy of this treatment method.

## Materials and methods

### General data

From January 2011 to January 2016, 67 patients with tuberculosis in the T4–6 segments who underwent one-stage posterior-only surgery by costotransverse debridement in combination with bone graft and internal fixation were selected. This study was approved by the Hospital Ethics Committee, and all patients provided written informed consent of participating in the study. There were 45 male cases and 22 female cases with an average age of 42.81 ± 9.05 years (range 25–62 years). Thirty-one patients visited due to the main complaint of back pain, 9 patients visited due to intercostal neuralgia, while the other 27 patients visited due to neurological impairment. According to the American Spinal Injury Association (ASIA) score system, the 27 patients consisted of 3 cases of grade B, 5 cases of grade C and 19 cases of grade D.

Surgical indications of patients in this study included: (1) there were spinal tuberculosis and paraplegia with ASIA score of grade A or B; (2) there were incomplete paraplegia with ASIA score of grade C or D, and imaging examinations showed that there was spinal compression; (3) the patients suffered from severe local pain, thus they were unable to walk, and effects of conventional analgesic drugs were poor; (4) there was severe or progressively aggravated kyphotic deformity; (5) there was spinal instability due to vertebral destruction^21^.

Inclusion criteria: (1). Spinal tuberculosis in T4–6 segments with surgical indications; (2). Patients aged older than 18 years; (3). Patients with bone destruction by tuberculosis mainly in T4–6 segments, and the destroyed segments were no more than 3 vertebral bodies. Exclusion criteria: (1). Patients with more than 3 destroyed vertebral bodies by tuberculosis, the focus of tuberculosis was beyond T4–6 segments; (2). Patients with distant gravitation abscess, and the scope of abscess was beyond T4–6 segments; (3). Patients combined with active tuberculosis; (4). Patients combined with other spinal disease may lead to biased prognosis evaluation.

### Preoperative examination and treatment

All the 67 patients underwent chest computed tomography (CT) to exclude active tuberculosis. All patients underwent thoracic X-ray, CT and Magnetic resonance imaging (MRI) examinations before surgery. Cobb angle of the kyphosis in the diseased segments was 18–63° with the average of 34.8 ± 10.9° by X-ray evaluation. Thoracic MRI and CT examinations suggested that there was bone destruction in the diseased vertebral bodies, and the involved intervertebral space was narrowed. Among them, 46 patients were combined with tuberculosis focus herniated into the spinal canal, leading to spinal stenosis of corresponding segments, and compression of the spinal cord or nerve. It had been confirmed by MRI that 42 patients were combined with paraspinal abscess formation. Distribution of the diseased segments included 16 cases of T4, 18 cases of T5, 8 cases of T6, 8 cases of T4–5, 10 cases of T5/6, and 7 cases of T4–6. There was no obvious abnormality in routine blood test for 45 patients, while 7 cases had mild anemia, and increased white blood cell count was found in 15 patients. Fifty-eight patients underwent the T-SPOT.*TB* test (an interferon (IFN)-γ release test), and the results were all positive, but this examination was not performed for the 9 cases who were treated before March 2014. Before operation, all the patients received intensive nutrition therapy. Twenty-seven patients had hypoproteinemia, thus amino acid and human albumin therapies were performed. Fifty-nine patients had continuous anti-tuberculosis treatment using isoniazid, rifampicin, pyrazinamide and streptomycin for more than 2 weeks, while 8 cases with severely progressive neurological impairment underwent surgical treatment after 1 week of intensive anti-tuberculosis treatment. At admission, the average erythrocyte sedimentation rate (ESR) of all patients was 91.30 ± 28.38 mm/h, and the average C-reactive protein (CRP) was 69.04 ± 35.33 mg/L (normal value of CRP:0–5.0 mg/L). Before the surgery, the average ESR was 40.91 ± 16.65 mm/h, and the average CRP was 25.07 ± 16.67 mg/L.

### Surgical method

All the patients were treated with one-stage posterior surgery by costotransverse debridement in combination with bone graft and internal fixation. After successful endotracheal intubation under general anesthesia, the patients were turned over for intraoperative neurophysiological monitoring. A longitudinal incision was made from the posterior median position with tuberculosis focus as the center. The lesions were exposed layer by layer, involving lamina, transverse process, costal transverse process costotransverse joints and little segment of the adjacent ribs, etc. First of all, bilateral pedicle screws were placed into both sides of the 2 vertebrae at the cephalic and caudal ends of the diseased vertebrae (Because tuberculosis severely damages the vertebral body, resulting in severe spinal instability and kyphotic deformity, in order to better restore the stability of the spine and correct the deformity, we choose to fix two stages at the head and tail of the diseased vertebrae). Debridement was performed on the side with severe destruction by tuberculosis or more abscesses based on CT and MRI results, while a temporary fixing rod was placed on the opposite side. After the temporary fixing rod was placed and locked tightly, costotransverse joints of the diseased segments were resected, and a 2–3 cm of adjacent rib was resected at the rib neck along the capitulum costae. Gelatin sponges were used for hemostasis. The tuberculosis focus was exposed towards ventral side along the bone structure of pedicle. Some pus or caseous necrotic tissue was harvested for pathological examination and *Mycobacterium* tuberculosis culture as well as drug susceptibility assay.

The lesions were debrided using nucleus pulposus forceps, bone rongeur, bone knife and curet. Debridement was performed until normal bone tissues were visible and there was petechial hemorrhage on the surface of cancellous bone. Irrigating was performed with hydrogen peroxide after debridement was completed, and the surgical area was soaked in povidone iodine solution for 5 min, then the lesion area and surgical area were rinsed using a small flushing gun with 3000 ml of saline. During the process, debridement can be performed from one side to the opposite side across the central line. Effective debridement can be completed for most patients, however, for patients with severe destruction in bilateral sides, the temporary fixing rod should be replaced and contralateral debridement should be performed once again.

For patients combined with neurological impairment or with compression-generating substances in the spinal canal, decompression with laminectomy in the diseased segments was performed before debridement to avoid spinal cord injury during the process. However, decompression was not required for patients without neurological impairment and spinal cord compression, and posterior structure of the diseased segments should be retained appropriately to improve postoperative stability.

After the irrigation was completed, the surgeons replaced the gloves, surgical gowns and surgical instruments, while a new large drape was placed. The intact pedicles of the diseased vertebrae can be fixed using short pedicle screws to improve the stability. Bilateral titanium rods were placed and kyphotic deformity was corrected. Bone defect area was slightly distracted. The resected rib or ilium was used for reconstruction of the anterior column for the focus based on the range of bone defect, and the anterior column was tightened slightly to stabilize the bone graft after reconstruction. Posterolateral fusion can be performed for patients with partial intact structure in the posterior side, which was not needed for patients with bilateral debridement. The wound was rinsed thoroughly, and gelatin sponges wrapped with isoniazid and/or streptomycin were placed in the bone graft area. Then, a drainage tube was inserted, and the wound was sutured to complete the surgery.

### Postoperative treatment

The patients were susceptible to postoperative wound infection, first-line antibiotics were usually applied after the surgery until examination of white blood cells was normal. The patients were conventionally treated with amino acids for 72 h. The drainage tube was removed when 24-h drainage volume was less than 50 ml. The patients should practice early ambulation after removing the drainage tube. Neurological rehabilitation should be performed for patients with neurological impairment guided by the rehabilitation department. The patients were discharged after condition was stable, and the patients should wear thoracodorsal brace for 3 months after discharge. Strengthened quadruple anti-tuberculosis therapy using isoniazid, rifampicin, pyrazinamide and streptomycin was performed for 3 months, and then streptomycin was discontinued. Ethambutol was added for continuous treatment for 9–15 months. ESR, CRP as well as liver and kidney functions were reviewed at postoperative 1, 3, 6, 12, 18 and 24 months until the patients were cured.

### Efficacy evaluation

Review was performed at postoperative 1, 3, 6, 12, 18 and 24 months as well as every 6 months after that. Conditions such as the presence of loosening and displacement of the internal fixation, as well as bone graft fusion, and changes of Cobb angle of thoracic kyphosis were evaluated. Clinical criteria for curing spinal tuberculosis of the patients were as follows: Follow-up was performed continuously for 2 years and symptoms of the patients disappeared; failure of internal fixation and sinus formation were not observed; bone fusion was confirmed by imaging examinations; ESR and CRP were normal for continuous 6 months. Clinical efficacy and recovery of neurological functions were evaluated by comparing Oswestry Dability Index (ODI, 0%: best functional state; 100%: worst functional state), Visual Analogue Scale (VAS, 0: no pain at all; 10: worst pain imaginable) score and ASIA score preoperative, postoperative and at the last follow-up. Odom’s criteria were used to evaluate the clinical outcomes of the 67 patients at the most recent follow-up.

### Statistical analysis

ESR, CRP, ODI score, VAS score and Cobb angle before surgery, after surgery and at the last follow-up were compared using paired *t*-test. The Rank sum test was used to compare the neurological grades before surgery and at the last follow-up. SPSS 21.0 (Chicago, IL, USA) software was used for statistical analysis. *P* < 0.05 was considered to be statistically significant, and all statistics were represented by mean ± standard deviation.

### Ethical consideration

The implementation of the research program complies with the "Declaration of Helsinki". And this study was approved by the Medical Ethics Committee of Honghui Hospital.

## Results

### Preliminary results after the surgery

All the 67 patients completed the surgery successfully. The average surgical time was 165.07 ± 27.93 min (ranged 110–220 min). The average intraoperative blood loss was 792.54 ± 297.15 ml (ranged 300–1500 ml). No patient suffered from severe complications such as large blood vessel or spinal cord injury. There was no cerebrospinal fluid leakage, no postoperative neurological deterioration and no significant surgery-associated complications. Postoperative pathological results confirmed that all the 67 patients suffered from tuberculosis. One patient developed ischemic necrosis of the skin margin because of not turning over in time postoperatively, thus the wound healed with delay and suture was taken out at postoperative day 27. Three cases treated with wound dressing daily and correction of hypoproteinemia due to hypoproteinemia-induced wound exudation after the surgery, then the wounds healed and sutures were taken out for the 3 patients at postoperative day 26, 28 and 31, respectively. Wound healing of all other patients was first intention, and sutures were taken out normally 14 days after the surgery. No infection was observed after the surgery, and the hospitalization stay of the patients was 10–35 days with the average of 15.61 ± 4.98 days.

### Follow-up results

**Figure 1:**
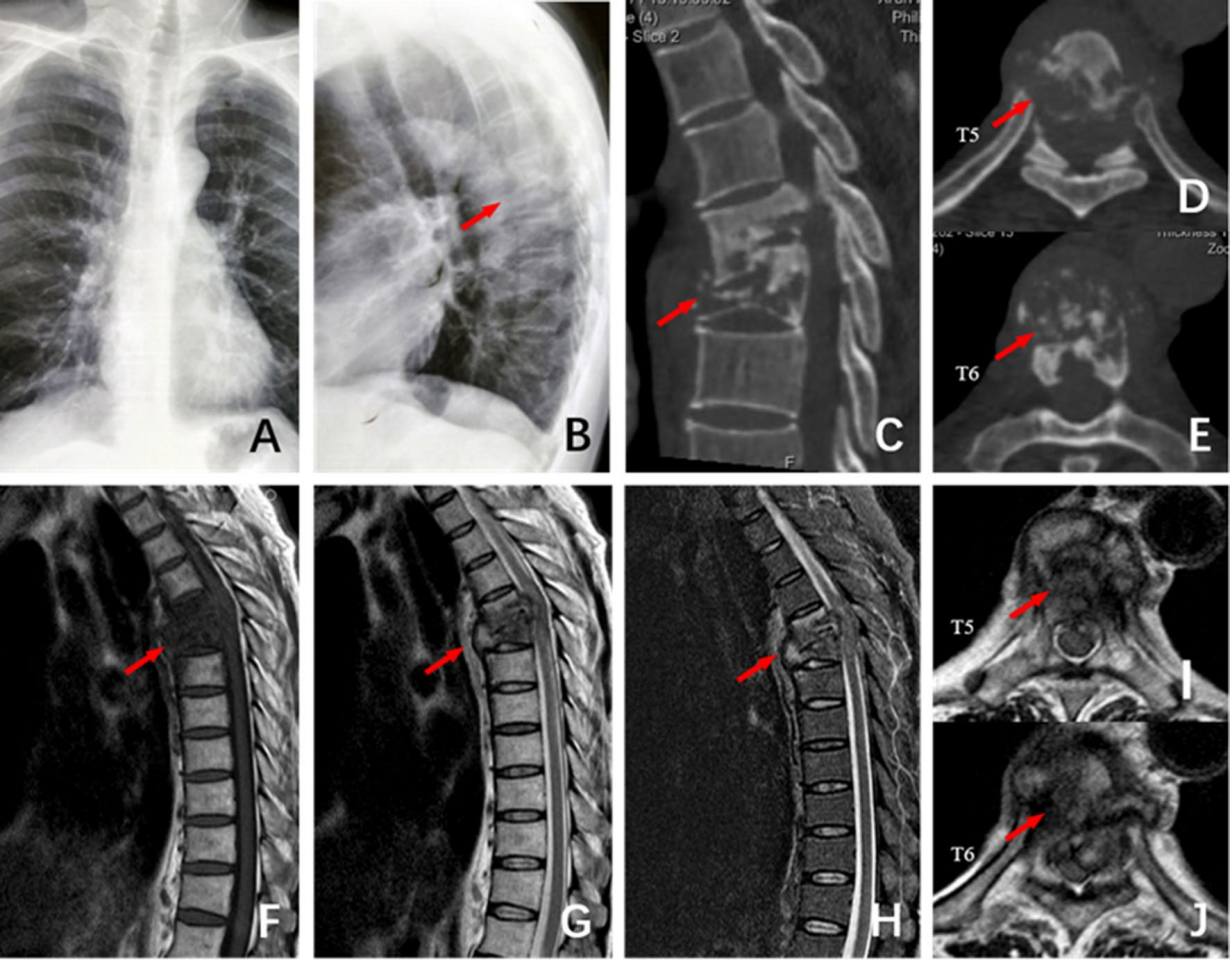
The patient, male, 51 years old, T5 ~ T6 spinal tuberculosis secondary to kyphosis deformity. (**A**) and (**B**) The frontal and lateral X-ray films before surgery showed vertebral destruction of T5 ~ T6 and Cobb Angle of 40°. (**C–E**) Preoperative CT showed severe damage to the anterior column of the T5 ~ T6 vertebral body and normal posterior column. (**F–J**): Preoperative MRI showed vertebral destruction of T5 ~ T6 and obvious compression of the spinal cord.

**Figure 2:**
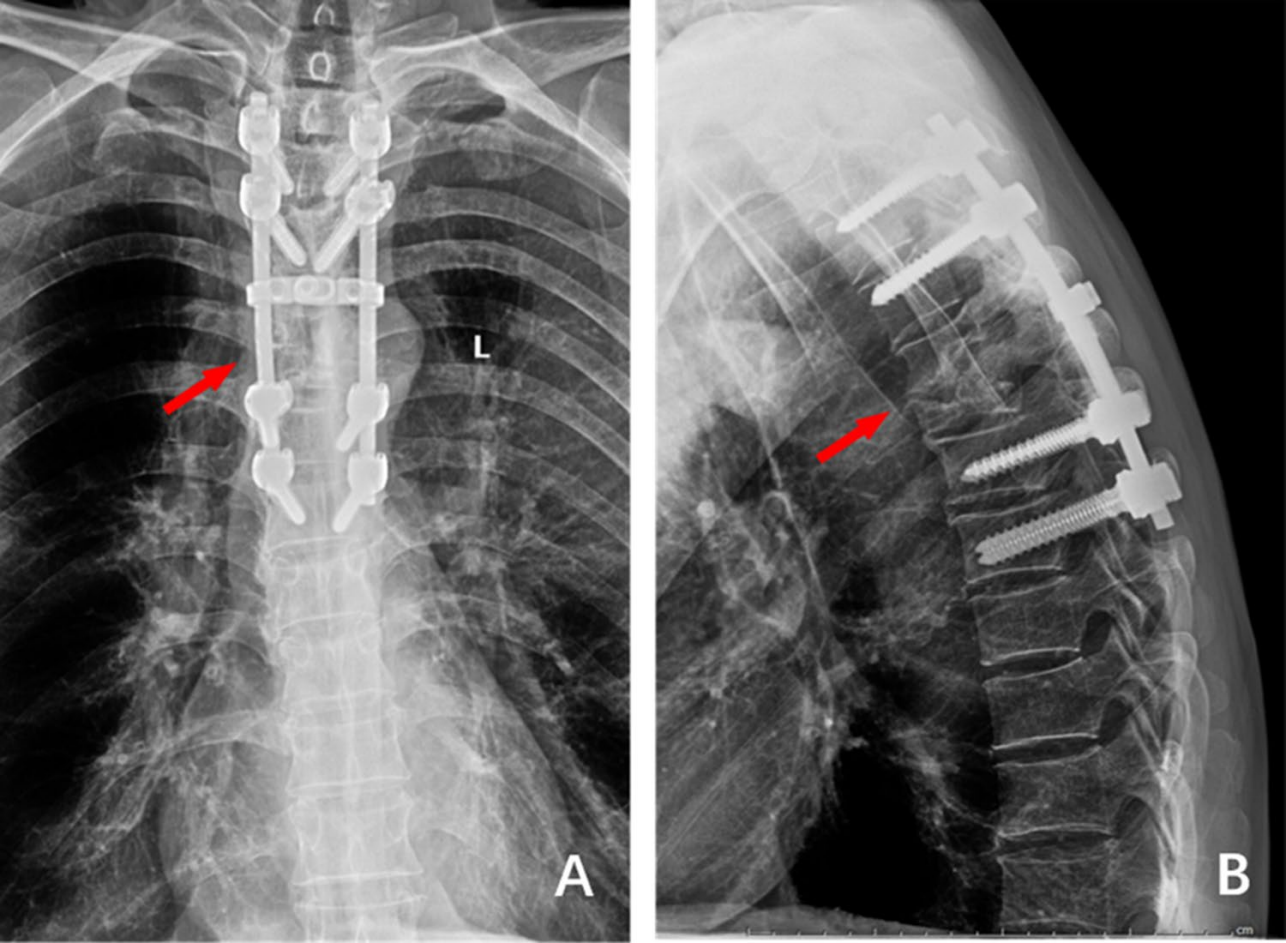
(**A**) and (**B**) 1 week after the operation, the frontal and lateral X-ray films showed that the internal fixation position was good, the deformity was corrected well, and The Cobb Angle was 19°.

**Figure 3:**
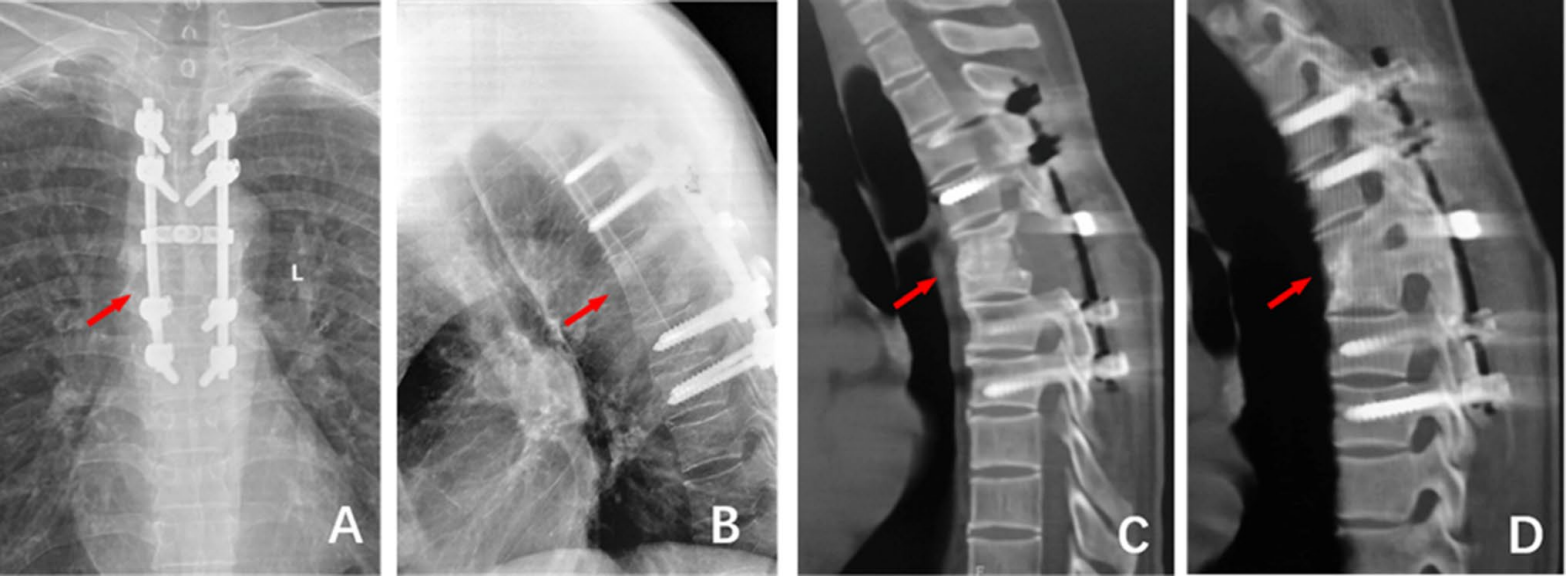
5 years after the operation, (**A**)and (**B**) The frontal and lateral X-ray films showed good internal fixation position, bone graft fusion, and Cobb Angle of 20°; (**C**) and (**D**): CT examination showed good internal fixation position and bone graft fusion.

All the 67 patients were followed up for at least 5 years. Clinical cure criteria of thoracic tuberculosis evaluated 2 years postoperative were satisfied for all patients, and tuberculosis recurrence was not observed in subsequent follow-ups. Review at postoperative 1 month showed that 11 cases (16.42%) complained of backache, thus they were administrated with topical analgesics, and the backache disappeared. ESR and CRP were returned to normal for all patients at 6 months follow-up. Among them, ESR and CRP were normal 1 month after the surgery for 45 cases (67.16%), which were normal 3 months after the surgery for 57 cases (85.07%). Moreover, increase in ESR and CRP did not occur in subsequent follow-ups. Compared with preoperative, the difference was statistically significant (P < 0.05) (Table 1). Seven cases had elevated transaminase during the follow-up period, but they recovered after treated with diammonium glycyrrhizinate, and no patient suffered from abnormal kidney functions. At the last follow-up, among the 27 patients combined with neurological impairment, neurological functions of 22 cases (81.48%) recovered completely. In addition, for the other 5 cases, 2 grade B patients improved to grade C and D respectively, 2 grade C patients improved to grade D, and 1 grade C patient remained unchanged. Compared with preoperative, the changes were statistically significant (P < 0.05) (Table 2). Bone fusion was confirmed in 67 patients during 6–9 months follow-up, and the average fusion period was 6.94 ± 1.40 months. During the follow-up period, no one patient had complications such as loosening, displacement and fracture of internal fixation. At the last follow-up, Cobb angle of the kyphosis was 11.3 ± 3.2°, which was statistically significant compared with that before the surgery (*P* < 0.05). At the last follow-up, ODI score was 11.2 ± 4.1, which was statistically significant improved compared with the preoperative score of 48.9 ± 15.4 (P < 0.05). VAS score at the last follow-up was 1.3 ± 1.1, which was statistically significant compared with the preoperative 7.1 ± 1.1 (P < 0.05) (Table 3). The improvement rates of ODI and VAS were 77.10% and 81.70%, respectively improvement rate = (final follow-up score – preoperative score) / (score at the best status—preoperative score) * 100%. Finally, 52 patients (91.2%) had good to excellent clinical outcomes, 3 patients (5.3%) had satisfactory results, and 2 patients (3.8%) had poor results. The preoperative and postoperative imaging data of typical case is shown in Figs. 1, 2, 3.

**Table 1:** Changes of ESR and CRP before and after the operation.

Time	ESR (mm/h)	Normal ESR (%)	CRP (mg/L)	Normal CRP (%)
Pre	50.91 ± 16.65	4.48(3/67)	34.07 ± 16.67	2.98(2/67)
Post 1 months	16.95 ± 3.16*	67.16(45/67)*	9.23 ± 2.54*	67.16(45/67)*
Post 3 months	13.16 ± 3.12*	85.07(57/67)*	3.42 ± 1.69*	85.07(57/67)*
Post 6 months	10.08 ± 2.39*	100(67/67)*	2.57 ± 2.03*	100(67/67)*

*, compare with preoperation *P* < 0.05.

ESR erythrocyte sedimentation rate, CRP C-reactive protein, Pre preoperative, Post postoperative.

**Table 2:** The condition of nerve functional restoration.

Pre ASIA grade	Patients (no.)	FFU* ASIA grade				
		A	B	C	D	E
A	0					
B	3			1	1	1
C	5			1	2	2
D	19					19
E	40					40

*compare with preoperation *P* < 0.05.

ASIA American Spinal Injury Association, Pre preoperative, FFU final follow-up.

**Table 3:** Changes of ODI and VAS and Cobb angle before and after the operation.

Time	ODI	VAS	Cobb angle (°)
Pre	48.9 ± 15.4	7.1 ± 1.1	34.8 ± 10.9
Post	45.2 ± 13.5	2.2 ± 1.5*	9.6 ± 2.8*
FFU	11.2 ± 4.1*	1.3 ± 1.1*	11.3 ± 3.2*

*, compare with preoperation *P* < 0.05.

ODI Oswestry Dability Index, VAS Visual Analogue Scale, Pre preoperative, Post Postoperative, FFU final follow-up.

## Discussion

The surgical approach has been controversial since the beginning of surgical treatment for spinal tuberculosis^19,22,23^, and the main issue is whether anterior surgery or posterior surgery should be used. Compared to anterior surgery, posterior surgery is easy to expose the lesions, which seems to be more advantageous. However, tuberculosis is usually in the anterior and middle column of the vertebral bodies, thus thorough debridement cannot be achieved by posterior surgery. “Thorough debridement” is of paramount importance in the treatment of spinal tuberculosis, thus direct anterior exposure and debridement are more suitable.

Due to the clinical specificity of spinal tuberculosis in T4–6 segments, the surgical treatment has become a challenge for spine surgeons. The treatment in T4–6 segments is different from spinal tuberculosis in the upper thoracic spines (T1-T3) as well as the mid-and lower thoracic spines (T7-T12). Tuberculosis in the T1-T3 segments can be debrided using lower anterior cervical approach that is familiar to surgeons, which can be combined with posterior surgery or be used alone for fusion and reconstruction^24^. Tuberculosis in the T7-T12 segments can be well exposed and debrided by conventional anterior transthoracic approach^25–27^. However, for T4–6, the anatomical position is deep, the surrounding tissues are complex, and there are many bony occlusions, such as sternal manubrium, clavicle, ribs and scapula. Moreover, the vertebral body is adjacent to the large blood vessel, aorta, thoracic duct and nerve tissues. Therefore, it is difficult to expose the lesions using anterior approach, and the surgical risk is high. Meanwhile, patients with tuberculosis are usually combined with destruction of bone structures, leading to kyphotic deformity, furthering increasing the difficulty of anterior surgery.

For T4–6 segments, the current anterior surgical approaches can be applied include subscapular transthoracic approach, mediastinal approach with sternotomy, clavicular approach, transsternoclavicular approach, and approach by resection of the manubrio-clavicular complex, etc.^10,14,24,28,29^. Although these surgical approaches can directly expose the lesions, and “thoroughly” debride the lesions as much as possible, they have the disadvantages of complex operations, unfamiliar to the spine surgeons, high surgical risk, and more complications including shoulder dysfunction, as well as injuries of vital organs such as recurrent laryngeal nerves, phrenic nerves, aorta and thoracic ducts, etc. In the meanwhile, trauma of anterior surgery is large, damage to the pulmonary appendices is great, and it is prone to pulmonary infection, atelectasis, pneumothorax and pleural effusion after surgery^24^. Thus, surgeons are extremely prudent to choose anterior approach for T4–6 segments due to the numerous shortcomings.

Multiple studies in recent years have demonstrated that posterior surgery is superior to anterior surgery for thoracic tuberculosis in reconstructing spinal stability and correcting kyphotic deformity. Meanwhile, anterior and posterior surgeries have similar cure rates for thoracic tuberculosis^6,12,17,30^. Wu et al.^12^ have conducted a retrospectively study for 394 patients with thoracic tuberculosis in 15 medical centers over 15 years. In their study, 73 patients undergo anterior surgery, 237 patients choose posterior surgery, and 84 cases use the combined surgery. The results have shown that clinical efficacy of posterior surgery alone is not different from anterior surgery as well as the combined surgery. Moreover, surgical time is short, and there is less blood loss in posterior surgery. In addition, posterior surgery is superior to anterior surgery in correcting kyphotic deformity and maintaining spinal stability. Li et al.^6^ have conducted a comparative study for the efficacies between anterior and posterior surgeries among patients aged over 65 years who have tuberculosis in T5-T12 segments. Their study results have suggested that posterior surgery is superior to anterior surgery, especially for patients with poor physical conditions. Wang et al.^30^ have conducted a systematical review for anterior and posterior surgeries in the treatment of thoracic and lumbar spinal tuberculosis recently. Their findings have shown that posterior surgery is better in correcting kyphotic deformity, and the pedicle system is used to compress the bone graft so that the bone graft is closely attached to the bone tissue after the focus is removed during the operation, which is conducive to fusion so as to obtain better spinal stability. In the present study, 67 patients were also treated with pedicle screw system to compress the anterior column reconstruction bone graft. All patients completed fusion during the follow-up period of 6–9 months, 81.48% of the patients with neurological impairment recovered completely, and the ODI score and VAS score increased by 77.10% and 81.70%, respectively. Our study results also confirmed the advantages of posterior surgery.

Compared to posterior surgery, an important purpose of anterior surgery is to “thoroughly debride the lesions”. Nevertheless, true thorough debridement cannot be achieved. Whatever surgical approaches cannot ensure that there is no *Mycobacterium tuberculosis* after debridement. Although posterior surgery is indirect debridement, it is also confirmed to be effective in debriding lesions in vital parts based on the main areas of spinal tuberculosis by the support of imaging examinations such as MRI and CT. Zhao et al. 18 have conducted a retrospective cohort study for 105 patients with thoracolumbar tuberculosis, and suggested that debridement via anterior or posterior approaches can effectively debride necrotic tissues surrounding the focus, and both of the two approaches have the same effects in debridement and curing ability of tuberculosis. Wang et al.^19^ have analyzed 51 cases with thoracolumbar tuberculosis, and they propose that spinal tuberculosis can be cured by fixation and fusion via posterior approach alone but there is no need of debriding lesion in the anterior side under the support of effective anti-tuberculosis drugs, and anterior debridement may be unnecessary. In our study, posterior debridement was performed for all patients. Rinsing with hydrogen peroxide was performed after debridement, and the surgical area was soaked in povidone iodine solution for 5 min. At last, the lesion area and surgical area were rinsed using a small flushing gun and 3000 ml of saline. After the process of debridement, there may still be *Mycobacterium tuberculosis* through posterior surgery, but most *Mycobacterium tuberculosis* was inactivated or washed away after the above treatments of rinsing and soaking, leading to better efficacy in posterior debridement. In our study, all the 67 patients obtained clinical cure, and there was no patient suffered from tuberculosis recurrence, confirming the effectiveness of debridement via posterior approach.

In our study, tuberculosis in T4–6 was put forward separately for the first time based on the clinical specificity of T4–6 segments, thus surgical treatment can be differentiated from other thoracic vertebrae (T1–3 and T7–12). This study focused on investigating the effectiveness and advantages of posterior surgery alone. However, this study had the disadvantage of small sample size. We believed that studies with large sample size, multi-center studies and even completely randomized studies would be performed in the future, which would be of great clinical significance for the treatment strategy of tuberculosis in T4–6 segments.

## Conclusions

In summary, effective clinical efficacy can be achieved for tuberculosis in T4–6 segments using one-stage posterior-only approach by costotransverse debridement in combination with bone graft and internal fixation. The posterior surgical method cannot only effectively accomplish debridement, obtain satisfactory clinical results, but also well correct kyphotic deformity and maintain it.

## Data Availability

The datasets generated and/or analyzed during the current study are available from the corresponding author on reasonable request.
